# Evaluation of Late Postoperative Pulmonary Function Following Lobectomy in Patients With Tubercular and Non-tubercular Lung Diseases

**DOI:** 10.7759/cureus.89419

**Published:** 2025-08-05

**Authors:** Rajeebshankar Karmakar, Raisa Enayet Badhan, Masnoon Ahmed Noor, Atik Ahmed Akond, Sutopa Halder Supti, Md Delwar Hossain

**Affiliations:** 1 Thoracic Surgery, Directorate General of Health Services, Dhaka, BGD; 2 Microbiology and Immunology, National Institution of Burn and Plastic Surgery, Dhaka, BGD; 3 Thoracic Surgery, National Institute of Diseases of the Chest and Hospital, Dhaka, BGD; 4 Paediatric Gastroenterology, Bangabandhu Sheikh Mujib Medical University, Dhaka, BGD

**Keywords:** lobectomy, non-tubercular lung disease, postoperative outcomes, pulmonary function, tubercular lung disease

## Abstract

Background: Pulmonary function testing, especially spirometry, is essential for assessing patients after pulmonary resection for tubercular and non-tubercular diseases. Tuberculosis (TB) remains a major cause of death globally, while other non-tubercular conditions such as lung abscess, bullous disease, and bronchiectasis also require lobectomy. This study aimed to compare late postoperative pulmonary function following lobectomy between TB and non-TB patients.

Methods: This cross-sectional study included 60 patients (30 TB, 30 non-TB) who underwent lobectomy at the National Institute of Diseases of the Chest and Hospital between January 2022 and June 2023. Pulmonary function was evaluated using spirometry, measuring forced vital capacity (FVC) and forced expiratory volume in one second (FEV_1_). The FEV_1_/FVC ratio was also calculated to assess the presence and severity of obstructive or restrictive lung patterns. Data were collected using a semi-structured questionnaire via face-to-face interviews.

Results: Among TB patients, 86.67% had postoperative forced expiratory volume in one second (FEV_1_) <2 L, compared to 60% in non-TB patients (p<0.05). Postoperative forced vital capacity (FVC) ≥2 L was found in 40% of TB and 43.33% of non-TB patients (p<0.05). Although FEV_1_/FVC improved in both groups, the difference was not statistically significant. Hospital stay, intraoperative bleeding, lobe involvement, and underlying disease showed significant differences between groups.

Conclusion: Non-TB patients demonstrated better postoperative pulmonary function than TB patients. These findings may guide perioperative planning and help reduce complications following lobectomy.

## Introduction

Tuberculosis (TB) remains one of the leading causes of death due to infectious diseases globally, posing a significant public health challenge in the 21st century [1,2]. In 2019, TB affected 10 million people and caused 1.4 million deaths, mostly in developing countries [3]. According to the WHO, there were 8.8 million TB cases globally in 2010 [4], and South Asia accounted for nearly 40% of the global burden in 2015 [1]. In 2011, Bangladesh was among the 22 highest TB-burden countries, with over 150,000 reported cases, predominantly from rural areas [4]. Surgical intervention in TB is indicated in cases of drug resistance, persistent sputum positivity, cavitary lesions, bronchopleural fistulas, multidrug-resistant TB (MDR-TB) with destroyed lung, massive hemoptysis, and bronchial stenosis [5]. TB treatment can significantly impact lung function, as seen in South African gold miners who showed reduced forced expiratory volume in one second (FEV_1_) and forced vital capacity (FVC) values even six months after treatment [5].

Non-tubercular pulmonary diseases, such as lung abscess, bullous disease, bronchiectasis, and aspergilloma, also require lobectomy. While some advocate for prophylactic resection in aspergilloma due to bleeding risk, others recommend surgery only after hemoptysis occurs. In bronchiectasis, surgery can prevent recurrent infections and hemoptysis, improving quality of life [6].

Pulmonary function tests, especially spirometry, are essential for preoperative and postoperative assessments. They assist in diagnosing disease, evaluating treatment response, and guiding surgical decisions [7,8]. Thoracoscopic lobectomy is associated with lower complication rates compared to thoracotomy, especially in patients with compromised pulmonary function [9]. However, loss of lung tissue may still lead to significant ventilatory impairment and cardiopulmonary complications [10].

Despite multiple studies on postoperative lung function following lobectomy, few have directly compared outcomes between tubercular and non-tubercular diseases. Given TB’s distinct pathophysiology and long-term lung damage, there is a need for focused research. This study aims to compare late postoperative pulmonary function - specifically FEV_1_, FVC, and FEV_1_/FVC - between patients undergoing lobectomy for tubercular and non-tubercular lung diseases.

## Materials and methods

This comparative cross-sectional study was conducted among patients diagnosed with either tubercular or non-tubercular pulmonary diseases who underwent lobectomy at the National Institute of Diseases of the Chest and Hospital (NIDCH). The study was carried out over 18 months, from January 2022 to June 2023, within the Department of Thoracic Surgery at NIDCH. Data were collected through a structured questionnaire, physical examination, and spirometric evaluation of pulmonary function. Spirometry was performed using the MIR Spirolab III (Medical International Research, Rome, Italy), a portable, computer-based device with a flow sensor accuracy of ±3% or ±50 mL and volume accuracy of ±3% according to ATS/ERS standards. The pulmonary function parameters measured included FVC, FEV_1_, and the FEV_1_/FVC ratio, which were used to assess the presence and severity of obstructive or restrictive ventilatory defects.

The study population consisted of patients admitted with indications for lobectomy due to tubercular or non-tubercular lung conditions and who fulfilled the predefined selection criteria. A convenient sampling technique was employed to recruit participants for the study, based on their availability and eligibility during the study period. The study's sample size consisted of a total of 60 participants, with 30 individuals allocated to each of the two respective groups.

Selection criteria

The inclusion criteria for this study were as follows: patients aged above 18 years who provided informed consent and were capable of performing spirometry. Eligible participants included those diagnosed with post-tubercular conditions, such as bronchiectasis, cavitary lesions, aspergilloma, and post-tubercular fibrosis, as well as those undergoing lobectomy for non-tubercular diseases, such as lung abscess, post-pneumonic bronchiectasis, bronchiectasis due to an impacted foreign body, pulmonary hydatid cyst, infected cyst, or multiple or giant bullae confined to a single lobe. Patients were excluded from the study if they had severe systemic illnesses such as uncontrolled diabetes mellitus, recent acute myocardial infarction, or hepatic, renal, or heart failure. Additionally, individuals undergoing lobectomy for pulmonary malignancy or those who were smear-positive for TB were excluded.

Data collection instrument

Semi-structured Questionnaire

Taking all relevant variables, at first, an English questionnaire was drafted. After necessary corrections and thorough checking, the English questionnaire was translated into Bangla.

Data processing and statistical analysis

After data collection, data processing and statistical analysis were carried out using Statistical Product and Service Solutions (SPSS, version 29; IBM SPSS Statistics for Windows, Armonk, NY). Continuous variables were presented as mean ± standard deviation or as median with interquartile range, depending on the distribution of the data. Categorical variables were expressed in terms of numbers and percentages. For univariate comparisons between the tubercular and non-tubercular groups, the chi-square test was used for categorical variables, while Student’s t-test was applied for continuous variables, as appropriate. All p-values were reported as two-sided, and a p-value of less than 0.05 was considered statistically significant.

Ethical considerations

Written informed consent was obtained from all participants after clearly explaining the purpose of the study, along with the potential benefits and risks associated with the procedure. Ethical approval for conducting the study was obtained from the Academic and Institutional Review Board (IRB) of the NIDCH, Mohakhali, Dhaka, Bangladesh.

## Results

Among all TB patients, the majority (50%) were in the age group of 31-50 years (n=15), followed by 26.8% in the 18-30 years group (n=11) and 13.6% in the 51-70 years group (n=4). Among non-TB patients, the majority (46.4%) were in the 18-30 years group (n=14), with 26.8% in both the 31-50 years group (n=8) and the 51-65 years group (n=8).

In terms of gender distribution, there were 22 male (73%) and eight female (26%) TB patients, while, among non-TB patients, 21 were male (70%) and nine were female (30%).

Regarding occupation, among TB patients, 21 (70%) were businessmen, 13 (20%) were housewives, two (6.6%) were students, and one (3.4%) was a teacher. Among non-TB patients, 26 were businessmen, two were housewives, one was a student, and one was a teacher. For smoking status, 10 TB patients (33.3%) were smokers, and 20 (66.7%) were non-smokers, whereas, among non-TB patients, nine (30%) were smokers and 21 (70%) were non-smokers.

In terms of diagnosis, among TB patients, 10 (46%) were diagnosed with aspergilloma, 13 (43%) had post-TB bronchiectasis, and three (11%) had other diagnoses. Among the non-TB group, 11 patients were diagnosed with bronchiectasis, and the remaining 11 were diagnosed with other conditions (Table 1).

**Table 1:** Comparison of the respondents according to diagnosis Statistical test used: chi-square test (χ²); TB: tuberculosis

Diagnosis	TB		Non-TB		Significance
	Frequency	Percentage	Frequency	Percentage	
Aspergilloma	14	46	0	0	Significant (p=<0.001, df=2, x^2^=25.803)
Bronchiectasis	13	43	11	36.7	
Others	3	11	19	63.3	
Total	30	100	30	100	

The study revealed that, among all TB patients, the majority (i.e., 62.1%) had lesions in the right lung, while 37.9% of them had lesions in the left lung. Among all non-TB patients, the majority (i.e., 53.3%) had lesions in the right lung, while 47.7% of them had lesions in the left lung. This finding is statistically not significant.

Among all TB patients, 15 had (i.e., 50%) lesion in the right upper lobe, nine had (i.e., 30%) lesion in the left upper lobe, three had (i.e.,10%) in right middle lobe, and rest of the three patients had (i.e., 10%) lesion in the left lower lobe. Among all non TB patients, seven had (i.e., 23.3%) lesion in the right upper lobe, four had (i.e., 13.4%) lesion in the right middle lobe, five had (i.e., 16.7%) in right lower lobe, seven had (i.e., 23.3%) in right middle lobe, and rest of the seven patients had (i.e., 23.3%) lesion in the left lower lobe (Table 2).

**Table 2:** Comparison of the tuberculosis (TB) and non-TB respondents according to the lobe of the lesion Statistical test used: chi-square test (χ²)

Site of lobe	TB		Non-TB		Significance
	Frequency	Percentage	Frequency	Percentage	
Right Upper	15	50	7	23.3	Significant (p=0.042, df=5, x^2^=9.902)
Right Middle	3	10	4	13.4	
Right Lower	0	0	5	16.7	
Left Upper	9	30	7	23.3	
Left Lower	3	10	7	23.3	
Total	30	100	30	100	

The study showed that, among all TB patients, the duration of the operative procedure was three or more than three hours in the case of 20 respondents (i.e., 67%), while the duration of the operative procedure was less than three hours in the case of the other 10 respondents (i.e., 33%). Among all non-TB patients, the duration of the operative procedure was three or more than three hours in the case of 20 respondents (i.e., 67%), while the duration of the operative procedure was less than three hours in the case of the other 10 respondents (i.e., 33%). This finding is statistically not significant.

The study showed that, among all TB patients, intraoperative bleeding was 500 mL or more in the case of 20 respondents (i.e., 66.7%), while the intraoperative bleeding was less than 500 mL in the case of the other 10 respondents (i.e., 33.3%). Among all non-TB patients, intraoperative bleeding was 500 mL or more in the case of seven respondents (i.e., 23.3%), while intraoperative bleeding was less than 500 mL in the case of the other 23 respondents (i.e., 76.7%) (Table 3).

**Table 3:** Comparison of the tuberculosis (TB) and non-TB respondents according to intraoperative bleeding Statistical test used: chi-square test (χ²)

Intraoperative Bleeding (in mL)	TB		Non-TB		Significance
	Frequency	Percentage	Frequency	Percentage	
< 500	10	33.3	23	76.7	Significant (p=0.001, df=58)
≥ 500	20	66.7	7	23.3	
Total	30	100	30	100	

The study explored that, among all TB respondents, postoperative hospital stay was more than 11 days in the case of nine patients (i.e., 30%), while the postoperative hospital stay was 11 days or less than that in the case of the other 21 respondents (i.e., 70%). Among all non-TB respondents, postoperative hospital stay was more than 11 days in the case of six patients (i.e., 20%), while the postoperative hospital stay was 11 days or less than that in the case of the other 24 respondents (i.e., 80%) (Table 4).

**Table 4:** Comparison of the tuberculosis (TB) and non-TB respondents according to postoperative hospital stay Statistical test used: chi-square test (χ²)

Postoperative Hospital Stays (days)	TB		Non-TB		Significance
	Frequency	Percentage	Frequency	Percentage	
≤ 11	21	70%	24	80%	Significant (p=<0.001, df=58)
> 11	9	30%	6	20%	
Total	30	100%	30	100%	

The study explored that, among all TB respondents, nine of them (i.e., 30%) developed postoperative complications, and 21 had (i.e., 70%) no complications. Among all non-TB respondents, six of them (i.e., 20%) developed postoperative complications, and 24 had (i.e., 80%) no complications (Table 5).

**Table 5:** Comparison of the tuberculosis (TB) and non-TB respondents according to postoperative complication Statistical test used: chi-square test (χ²)

Postoperative Complication	TB		Non-TB		Significance
	Frequency	Percentage	Frequency	Percentage	
Yes	9	30	6	20	Not significant (p=0.371, df=1, x^2^=0.80)
No	21	70	24	80	
Total	30	100	30	100	

The postoperative FEV_1_ of those TB patients was more than 2 L in the case of four patients (i.e., 13.33%), and less than 2 L in the case of 26 respondents (i.e., 86.67%). Among all non-TB respondents, the postoperative FEV_1_ of those non-TB patients was more than 2 L in the case of 12 patients (i.e., 40%), and less than 2 L in the case of 18 respondents (i.e., 60%) (Table 6).

**Table 6:** Comparison of the tuberculosis (TB) and non-TB respondents according to postoperative FEV1 Statistical test used: chi-square test (χ²)

Postoperative FEV_1_ (in liter)	TB		Non-TB		Significance
	Frequency	Percentage	Frequency	Percentage	
≥ 2	4	13.3	12	40	Significant (p=0.043, df=58)
< 2	26	86.67	18	60	
Total	30	100	30	100	

This study showed that, among all TB respondents, the postoperative FVC of those TB patients was more than 2 L in the case of 12 patients (i.e., 40%), and less than 2 L in the case of 18 respondents (i.e., 60%). Among all non-TB respondents, the postoperative FVC of those TB patients was more than 2 L in the case of 17 patients (i.e., 43.33%), and less than 2 L in the case of 13 respondents (i.e., 56.67%) (Table 7).

**Table 7:** Comparison of postoperative forced vital capacity (FVC) (in liters) between tuberculosis (TB) and non-TB patients Statistical test used: chi-square test (χ²)

Postoperative FVC	TB Patients		Non-TB Patients		Significance
	Frequency	Percentage	Frequency	Percentage	p=0.024, df=58 (Significant)
≥ 2 liters	12	40%	17	43.33%	
< 2 liters	18	60%	13	56.67%	
Total	30	100%	30	100%	

In this study, among all TB respondents, the postoperative FEV1/FVC of 30 TB patients was normal in the case of 18 patients (i.e., 60%) and below normal in the case of the other 12 respondents (i.e., 40%).

Among all 30 non-TB respondents, the postoperative FEV1/FVC of those non-TB patients was normal in the case of 24 patients (i.e., 80%), and the preoperative FEV1/FVC was below normal in the case of the other six respondents (i.e., 20%) (Table 8).

**Table 8:** Comparison of the tuberculosis (TB) and non-TB respondents according to the preoperative and postoperative FEV1/FVC Statistical test used: chi-square test (χ²)

Postoperative FEV_1_/FVC (in percentage)	TB		Non-TB		Significance
	Frequency	Percentage	Frequency	Percentage	
≥ 70	18	60	24	80	Not significant (p=0.169, df=58)
< 70	12	40	6	20	
Total	30	100	30	100	

## Discussion

This cross-sectional study compared postoperative outcomes of lobectomy between patients with tubercular and non-tubercular lung diseases admitted to the NIDCH from January 2022 to June 2023. A total of 60 patients (30 in each group) were selected using a convenient sampling technique, and data were collected through face-to-face interviews using a semi-structured questionnaire. There were no missing data, and statistical analysis was performed using SPSS.

Among tubercular patients, the mean age was 36.57 years, with a male predominance (73.3%), and most were non-smokers. Lesions were primarily located in the upper lobe of the right lung. Postoperative complications, such as bleeding and air leaks, were noted in one-third of the TB group. TB causes extensive lung inflammation, compromising vascular and airway integrity, leading to bleeding, air leaks, and complications such as bronchiectasis [2,11,12]. Prolonged surgical duration (≥3 hours), significant intraoperative bleeding (≥500 mL), and longer hospital stays (>11 days) were common, attributed to the structural lung damage caused by TB and systemic factors, such as immunosuppression [3,11,13,14]. Despite these challenges, there was no postoperative mortality, consistent with findings by Amorim et al. [2] and Pomerantz et al. [15], who reported a 3.3% operative mortality in MDR-TB cases, mainly linked to previous pneumonectomy and extensive fibrosis. Park et al. [16] also highlighted the role of low serum albumin and intraoperative bleeding in postoperative respiratory failure.

In the non-tubercular group, the mean age was 36.83 years, with 70% being male and most being non-smokers. Similarly, lesions were mostly located in the right upper lobe. However, the operative time, blood loss, and hospital stay were generally lower compared to the TB group. Only one-fifth of these patients developed postoperative complications, with no mortality. Ludwig et al. [17] reported pulmonary complications in 19-27% of non-TB patients, depending on supportive interventions. Babatasi et al. [18] reported a 4% operative mortality in aspergilloma surgery, with complications such as bleeding, prolonged air leak, respiratory failure, and empyema.

Pulmonary function outcomes showed that 86.67% of TB patients had postoperative FEV_1_ <2 L, and 60% had FEV_1_/FVC ≥70%. In contrast, 60% of non-TB patients had FEV_1_ <2 L, and 80% had FEV_1_/FVC ≥70%, suggesting relatively better lung function in non-TB patients. However, the difference in FEV_1_/FVC was not statistically significant.

The study acknowledges several limitations. It was conducted at a single center, limiting generalizability. The sample size was small, partly due to COVID-19-related challenges, and long-term follow-up was not performed, preventing assessment of long-term postoperative outcomes and complications.

## Conclusions

A significantly higher proportion of TB patients had postoperative FEV_1_ values below 2 L compared to non-TB patients, indicating greater challenges in lung function recovery. Although postoperative FEV_1_/FVC ratios increased in both groups, the change was not statistically significant. Additionally, postoperative FVC values showed significant variation between TB and non-TB patients.

Factors such as hospital stay duration, intraoperative bleeding, and the specific lung lobe involved also significantly influenced surgical outcomes. These findings highlight the need for tailored postoperative care and follow-up strategies for each patient group. By recognizing the distinct challenges faced by TB and non-TB patients, clinicians can better optimize management approaches, ultimately improving postoperative pulmonary function and enhancing patients’ quality of life.
